# Action Evaluation Is Modulated Dominantly by Internal Sensorimotor Information and Partly by Noncausal External Cue

**DOI:** 10.1371/journal.pone.0034985

**Published:** 2012-05-01

**Authors:** Takao Fukui, Hiroaki Gomi

**Affiliations:** 1 NTT Communication Science Laboratories, Nippon Telegraph and Telephone Corporation, Morinosato, Atsugi, Kanagawa, Japan; 2 CREST, Japan Science and Technology Agency, Honcho, Kawaguchi, Saitama, Japan; Centre national de la recherche scientifique, France

## Abstract

Previous studies demonstrated that human motor actions are not always monitored by perceptual awareness and that implicit motor control plays a key role in performing actions. In addition, appropriate evaluation of our own motor behavior is vital for human life. Here we combined a reaching task with a visual backward masking paradigm to induce an implicit motor response that is congruent or incongruent with the visual perception. We used this to investigate (i) how we evaluate such implicit motor response that could be *inconsistent* with perceptual awareness and (ii) the possible contributions of reaching error, external visual cues, and internal sensorimotor information to this evaluation. Participants were instructed, after each trial, to rate their own reaching performance on a 5-point scale (i.e., smooth – clumsy). They also needed to identify a color presented at a fixation point that could be changed just after the reaching start. The color was linked to the prime-mask congruency (i.e., congruent-green, incongruent-blue) in the practice phase, and then inconsistent pairs (congruent-blue or incongruent-green) were introduced in the test phase. We found early trajectory deviations induced by the invisible prime stimulus, and such implicit motor responses are significantly correlated with the action evaluation score. The results suggest the “conscious” action evaluation is properly monitoring online sensory outcomes derived by implicit motor control. Furthermore, statistical path analyses showed that internal sensorimotor information from the motor behavior modulated by the invisible prime was the predominant cue for the action evaluation, while the color-cue association learned in the practice phase in some cases biases the action evaluation in the test phase.

## Introduction

We believe that we perform our daily actions with continuous access to conscious awareness, but previous studies have demonstrated that this is not true and that we are actually aware “only of the tip of the action iceberg” (e.g., [1]). Two examples of this dissociation between movement and awareness stand out. First, several studies [2]–[5] have shown that a stimulus that cannot be perceived consciously influences motor responses in a visual backward masking paradigm. This paradigm is based on a psychophysical procedure in which conscious perception of a briefly presented stimulus (i.e., the prime) is cancelled by masking another ‘mask’ stimulus (e.g., [6]–[8]). Second, participants can automatically adjust their motor behavior for a target shift during their movements even when they are not consciously aware of the target location change (e.g., [9], [10]).

These studies have demonstrated the dissociation between perceptual awareness and implicit motor control. But if we can act without perceptual awareness, one might ask how we recognize and perceive the consequences of such actions (i.e., an experience of action, e.g., [11]). Johnson and Haggard [12] demonstrated a dissociation between motor awareness and perceptual awareness. Specifically, they found that participants can reproduce the spatial details of a visuomotor adjustment in double-step pointing, regardless of perceptual awareness of a target shift when the target location is actually shifted. In our daily life, a dramatic example of motor behavior’s betrayal of conscious awareness is stepping on a stopped escalator. Fukui, Kimura, Kadota, Shimojo, and Gomi [13] compared the properties of motor behavior toward a stopped escalator with those toward a moving escalator and toward a wooden stairs that mimicked the stopped escalator. We found that, just after one steps onto a stopped escalator (not a wooden stairs), one experiences a forward sway of the upper body subconsciously driven by a habitual motor program with some kind of odd sensation, despite a suitable action intention and full awareness of the environmental situation (i.e., the escalator is stopped). Fukui et al. [13] also, in a sense, demonstrated a dissociation between perceptual awareness and motor awareness (i.e., motor awareness *against* perceptual awareness, rather than motor awareness without perceptual awareness). We further found the participants indeed feel an odd sensation in such situation, while Johnson and Haggard [12] did not report any unusual sensations associated with visuomotor adjustment without perceptual awareness of target shift. We suggest that unusual sensations emerge depending on whether a monitoring system does or does not detect conflict between action intention and motor outcome (cf. [14]).

If the brain indeed evaluates the consequent sensation induced by implicit motor control, we might ask what information is critical for this evaluation. In particular, online sensorimotor information, final motor error, and associated sensory information might all contribute.

Recent studies stress that prediction plays a key role in the emergence of motor awareness [15]–[18] as well as in recent motor control theory (e.g., [19]–[21]). According to these studies, appropriate monitoring of the sensorimotor congruence between prediction (computed by a forward internal model) and actual sensory feedback is crucial for action evaluation. Alternatively, the action evaluation could be inferred retrospectively (e.g., [22], [23]). According to this hypothesis, even an external cue irrelevant to motor behavior itself could be used in action evaluation. Within a framework of these two theories (see also [24]), further questions about the action evaluation arise as described below. Tackling these questions is important for revealing the mechanism of performance improvement [25] and elucidating the relationship between sensorimotor integration and the self-specifying process [26].

1. When we evaluate our own motor behavior induced against perceptual awareness, could online deviation by implicit motor control be monitored and used in the matching process of sensorimotor congruence (cf. [27])? Or is final motor error a predominant clue for such a matching process (cf. [28])?
2. When an external cue associated with motor behavior happens to conflict with the outcome induced by implicit motor control, does the external cue, which had previously a perfect association with one’s own motor behavior but has now an opposite association, mistakenly bias the action evaluation?

To examine these questions, we combined a reaching task with a visual backward masking paradigm. A reaching task is more appropriate than a simple reaction time task for revealing a dynamic information process [29]. In this task the reach trajectory is specified by a combination of mask and target stimuli, but the mask was preceded by an invisible prime. The prime induces implicit motor control in the opposite direction to the reach target, i.e., against perceptual awareness of the target [29]–[33]. Furthermore, the prime-mask congruency was linked to the color of a fixation point (i.e., congruent-green, incongruent-blue) in a practice phase to associate action evaluation with the color, and additional new pairs (either congruent-blue or incongruent-green) were introduced in a small percentage in a test phase in two different participant groups (i.e., an incongruent-green group and a congruent-blue one). Here our focus is to examine whether, under such a condition, participants are able to appropriately monitor their action using online sensorimotor information or whether they evaluate their action using final reaching error and/or associated sensory information (i.e., the color of a fixation point). In simple terms, what information do participants rely on more for the action evaluation in the test phase when they happen to perform the trials in the new condition (congruent-blue or incongruent-green), which now conflicts with the association learnt in the practice phase? It is noteworthy that the external color cue itself originally has no causal relationship with one’s own motor behavior induced by the prime-mask congruency and is associated with it during the practice phase.

The results show that monitoring online sensorimotor information as specified by the masked prime plays a predominant role in action evaluation. The action consequence (i.e., endpoint error) partly contributes, but its effect on action evaluation is much smaller than the effect of online sensorimotor information. Furthermore, statistical path analyses reveal some involvement of a noncausal external color cue linked to prime-mask congruency associated in the practice phase, suggesting some visual cue effect for the action evaluation.

## Materials and Methods

### Participants

Eight males and 18 females (20–40 years of age, mean age = 28.3±6.3 years) participated in the experiment. All participants reported normal or corrected-to-normal vision and none of them had any motor or sensory abnormalities. They gave written informed consent to participate in the study, which was approved by the NTT Communication Science Laboratories Research Ethics Committee. All participants were right-handed. This experiment consisted of two phases: a color-action association learning phase (practice phase) and a test phase. Considering the aim of this study, we excluded the following participants from the analyses: 1) Those who (two participants) noticed the existence of the prime stimulus during the experiment due to violation of the instructions. For example, they were fixating not the fixation point but prime-mask stimuli themselves. 2) Those who (three participants) showed no (or little) effect of implicitly driven motor behavior (by invisible prime) on action evaluation in the practice phase. As for the latter exclusion case, since we wanted to examine what information (online sensorimotor information, endpoint error, or an external cue) is the more critical clue for the action evaluation in the test phase, we needed an association between color and action in the practice phase, which means a higher score in the congruent-green condition and a lower one in the incongruent-blue condition (see Procedure and Result sections for details).

In the test phase, we divided the participants into two groups: an incongruent-green group (three males and eight females, mean age = 28.3±6.7 years) and congruent-blue group (three males and seven females, mean age = 27.6±6.9 years) as described below in detail.

### Apparatus

**Figure 1:**
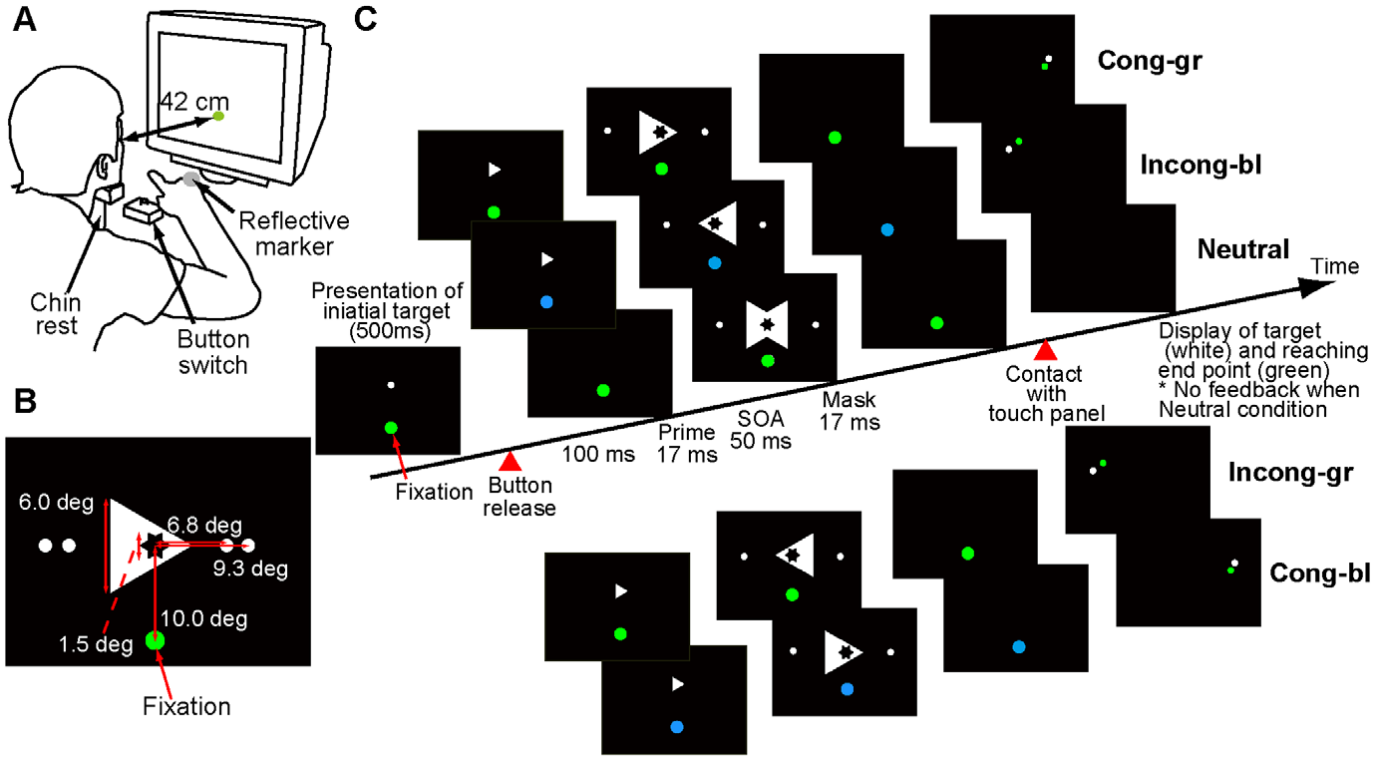
Experimental setup and protocol. (A) Configuration of the experimental apparatus. Participants rested their heads on the chin rest and were required to make reaching movements from the button switch to the target displayed on the monitor while they watched the fixation point. The motion of the reflective marker was recorded by the motion capture system as the kinematics of the reaching movement. (B) Visual stimulus layout. The diameter of the fixation point participants were asked to watch during the trials was 0.85 deg (shown as a green filled circle). Right- or left-pointing prime and mask stimuli were regular triangles whose sides were 1.5 and 6.0 deg, respectively. The centroid of the prime triangle was located at 10 deg above the fixation point in the vertical plane. Possible targets (white filled circle, diameter of 0.6 deg) were located at 6.8 and 9.3 deg horizontally from the centroid on each side, respectively. (C) Time sequence of the visual stimuli (prime-mask combination) in each condition. While the fixation point remained green in the congruent(incongruent)-green and neutral conditions after the button release, the color of the fixation point was switched from green to blue in the incongruent(congruent)-blue conditions at the same timing of prime onset. The outer contour of the prime stimuli fit exactly within the inner contour of the cutout of the mask triangles. Except for trials in the neutral condition, either the right- or left- target point (white filled circle, diameter: 0.6 deg) reappeared according to the direction of the mask stimulus, and feedback about the reaching endpoint (green filled circle, diameter: 0.6 deg) was also provided after the reaching movement (i.e., contact with touch panel).

Participants viewed the CRT display (60 Hz) from a distance of approximately 42 cm, with their head movement restricted by a chin-rest (see Fig. 1A).

Right- or left-pointing prime and mask stimuli were regular triangles whose sides were 1.5 and 6.0 degrees, respectively. The centroid of the prime triangle was located at 10 deg above the fixation point in the vertical plane. The outer contour of the prime stimuli fit exactly within the inner contour of the cutout of the mask triangles. A neutral mask was formed from the superimposition of the two triangles (see Figs. 1B and 1C).

The prime triangle was presented 100 ms after a button had been released at the start position. It was presented for 17 ms. The prime-mask stimulus-onset asynchrony (SOA) was set at 50 ms and mask presentation was 17 ms (see Fig. 1C). Right and left target points were simultaneously presented with the mask stimulus in each near or far condition (near: 6.8 deg, far: 9.3 deg from the center of the cutout, see Fig. 1B). The mask stimulus was also a cue stimulus for reaching direction.

The reaching end position was recorded with a touch panel (Keytec. Inc). The right-hand position (monitored by a reflective marker placed on the back of the hand around the bottom of the ring finger) was recorded with a three-dimensional motion capture system (ProReflex, Qualisys, Sweden) at a frequency of 250 Hz.

### Procedure

Before a reaching task, we needed to obtain the perceptual threshold of prime stimulus intensity so that participants would not be able to recognize the prime triangle in the main experiment. For this purpose, we had participants perform a prime identification task using a Bayesian adaptive psychometric procedure, QUEST [34]. Participants were requested to report the direction of the prime triangle in the two-alternative choice manner for prime (right, left) and mask (right, left) combinations randomly selected (80 trials). QUEST is a procedure for running each trial at whatever signal strength would contribute most to minimizing the variance of the final threshold estimate (see also [35]). Such a procedure combines the experimenter's prior knowledge (tGuess = −1, tGuessSd = 5.0, beta = 3.5, delta = 0.01, gamma = 0.5 in QUEST software of Psychophysics Toolbox [36]) and the observer's responses (i.e., right or left) in past trials in choosing the signal strength for the next trial, and, in the end, estimating threshold. After each response, a Gaussian probability density function is updated by Bayes’ rule. Each trial is placed at the current maximum-likelihood estimate of the threshold. The threshold of each participant was set at 51%, which is rather conservative compared to the threshold generally assigned (i.e., about 70%). We confirmed that the final threshold estimates appropriately converged. With this setting, we assumed that the performance level of each participant would be almost chance level. After this task, we also confirmed, from participants’ reports (cf. [37]), that participants could not identify the direction of the prime stimulus at all.

Next, participants performed the reaching task using the backward masking paradigm in a darkened room. As shown in Figs. 1A and 1B, the participants were asked to place their heads on a chin-rest and to fixate a green fixation point (diameter of 0.85 deg). Each trial started by participants’ pressing a button placed at the start position with the index finger. The initial reaching target (white dot, diameter of 0.6 deg) was presented 10 deg above the fixation point for 500 ms from the start time of the button press, and participants were instructed to intend to reach for this initial target. After the target point disappeared, two beeps at intervals of 200 ms were provided. Participants were required to start moving (i.e., release the button) at the second “go” beep and reach for the target point. When a neutral mask stimulus was presented, the participants were required to reach for the initial target corresponding to the center of the cutout. The participants had to correct their reaching movements according to the direction of the mask triangle and reach for the point consistent with the mask direction out of the two presented target points when a right- or left- pointed triangle mask was presented (the mask stimulus was thus also a cue stimulus for reaching direction as mentioned above). After the reaching movements, the target point and reaching endpoint were shown in white and green, respectively, except in the neutral condition. There was no feedback error information when the neutral condition was applied. The participants were instructed not to shift their gaze to the triangle (mask) stimulus or the target point but to keep watching the fixation point during the trial.

After each trial, except those in the neutral condition, the participants were requested to answer the following two questions: Did the fixation point color remain green or did it switch from green to blue (the color identification task)? To what extent did you appropriately perform the movement according to your intention of reaching for the target based on the stimulus (mask) direction? The second question was reported on a 5-point scale (i.e., 5 - very smooth; 1 - very clumsy).

The reaching task consisted of two phases: a color-action association learning phase (practice phase) and a test phase. In the practice and test phases, 384 and 192 trials were completed, respectively. A short break was inserted every 48 trials. In the practice phase, there were three conditions as shown in Fig. 1C: 1) congruent-green (50%), in which the prime direction was congruent with the mask one [prime-mask combinations of right-right (RR) and left-left (LL)] and the color of fixation point remained green; 2) incongruent-blue (33%), in which the prime direction was incongruent with the mask one [prime-mask combinations: right-left (RL) condition and left-right (LR)] and the color of fixation point was switched to blue; 3) neutral (17%), in which neutral mask triangle without the prime was presented and the color of the fixation point remained green. In the test phase, an additional color-stimuli combination condition, either incongruent-green or congruent-blue, was inserted in addition to the congruent-green, incongruent-blue, and neutral conditions (see Fig. 1C). The rate of each condition was as follows. Congruent-green condition 50%, incongruent-blue condition 25%, incongruent-green or congruent-blue condition 8%, and neutral condition 17%. Trial order was pseudo randomized in both phases. The participant group in the practice phase was divided into two groups in the test phase: an incongruent-green group, in which the incongruent-green condition was inserted, and a congruent-blue group, in which the congruent-blue condition was inserted.

### Data Processing and Analysis

The hand position data were temporally aligned with respect to the button release and were filtered offline using a fourth-order Butterworth filter (double-sided) with a cutoff frequency of 10 Hz. The velocity was calculated from three-point numerical time differentiations of the filtered position data. Our main interest is the effect of the prime direction on motor behavior, so we analyzed the following data pooled from both target positions (i.e., near and far conditions). To quantify the effect of the prime stimulus on motor behavior, we calculated the mean velocity of a 100-ms-time window from 200 to 300 ms after prime onset in each condition. We adopted the mean X-velocity as the index of the prime effect because velocity is more suitable for detecting the transient behavioral changes than position (trajectory). The response onset to the prime stimulus was defined as the time from which the velocity differences between RR (LL) and LR (RL) continuously exceeded a threshold value of 30 mm/s for at least 40 ms in a window between 150 and 300 ms after the prime onset. The threshold value approximately corresponded to 2 SDs of the velocity during a period of 0–50 ms after the button release (cf. [38]). Endpoint error was calculated as the Euclidean (linear) distance in two-dimensional space (on the vertical plane) between the endpoint of the reaching movement recorded with the touch panel and the target position (see Figs. 1A and 1C). Mean endpoint error in each condition was averaged by the sum of each trial’s endpoint error in each condition. Repeated measures ANOVAs were applied to these mean values. Specifically, the prime direction (right, left) and mask one (right, left) were within-participant factors for velocity, and mask direction (right, left) and conditions [congruent-green, incongruent-blue] were within-participant factors for the endpoint error and action evaluation score in the practice phase. In the test phase, the prime direction (right, left) and condition [congruent-green, incongruent-blue, incongruent-green (or congruent-blue)] were within-participant factors for the velocity, and the mask direction (right, left) and conditions [congruent-green, incongruent-blue, incongruent-green (or congruent-blue)] were within-participant factors for the endpoint error. Tukey's HSD procedure was used for post-hoc comparison of means (alpha level  = .05). As for the action evaluation score, we are interested in whether the action evaluation is modulated by the color cue itself, so we performed planned *t* tests on the mean evaluation scores for incongruent-blue and incongruent-green conditions in the incongruent-green group and on those for congruent-green and congruent-blue conditions in the congruent-blue group.

Finally, to identify the information used in the action evaluation, we introduced path analyses. We have the assumption that online corrections induced by the invisible prime stimulus and/or consequent endpoint error are potential candidates that could affect the action evaluation in the practice phase. In the test phase, the color cue would inferentially contribute to the evaluation of one’s own action. The color cue variable introduced in the test phase was nominal scale, so we used a dummy variable for the analysis; that is, blue was transformed to 0 and green was transformed to 1. For each variable, we calculated the variance inflation factor (VIF), which, as suggested by Myers [39], should be less than 10 to avoid multicollinearity problems. We focused on the standardized path coefficients between variables. Standardized path coefficients indicate the relative effect of variables within the model.

## Results

As mentioned above, we excluded participants who showed no significant different action evaluation scores between congruent-green and incongruent-blue conditions in the practice phase (i.e., the second exclusion case in the Participants section). After the experiment, participants reported whether they had not noticed the existence of the prime stimulus during the experiment.

### Practice Phase

The mean correct rate in the color identification task in the practice phase was 97.4% (SD = 2.4%), indicating participants performed the color discrimination task nearly perfectly. Only correct trials in the color identification task were analyzed.

**Figure 2:**
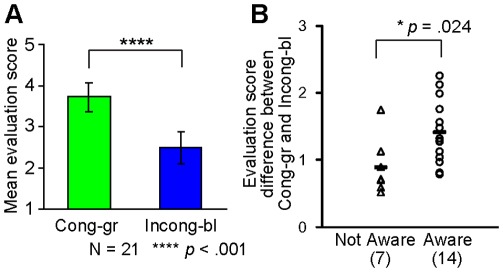
Action evaluation of one’s own motor behavior. (A) Mean evaluation scores in the congruent-green and incongruent-blue conditions of the practice phase. Error bars indicate the SDs of the evaluation scores between participants. (B) Difference of evaluation scores between the congruent-green and incongruent-blue conditions of the practice phase. Triangles denote the data for each participant of the “not aware” group, those who did not report an association between their own performance and the color cue. Circles denote that of the “aware” group, those who reported an association between their own performance and the color cue.

The 21 participants showed a significantly higher congruent-green score than incongruent-blue one, as revealed by each participant’s *t* test. Mean evaluation scores of congruent-green and incongruent-blue conditions are shown in Fig. 2A (*t*(20) = 11.03, *p*<.001, *r* = .93). Out of these participants, 14 reported after finishing the practice phase that they felt smoother performance for the green cue and clumsier performance for the blue one. Seven participants reported that they felt no difference between the green and blue cues even though they showed a significant difference of action evaluation scores between the congruent-green and incongruent-blue conditions. The participants who were aware of the association between their own performance (induced by the prime-mask combination) and the color cue (i.e., the “aware” group in Fig. 2B) showed a significantly larger difference of mean evaluation scores between the congruent-green and incongruent-blue conditions than those who did not notice such an association (“not aware” group in Fig. 2B, *t*(19) = −2.454, *p* = .024, *r* = .49). As mentioned in the introduction, the color cue itself essentially has no causal link with one’s own motor behavior, so such association emerged through trials in the practice phase.

#### Invisible prime affects online motor control

**Figure 3:**
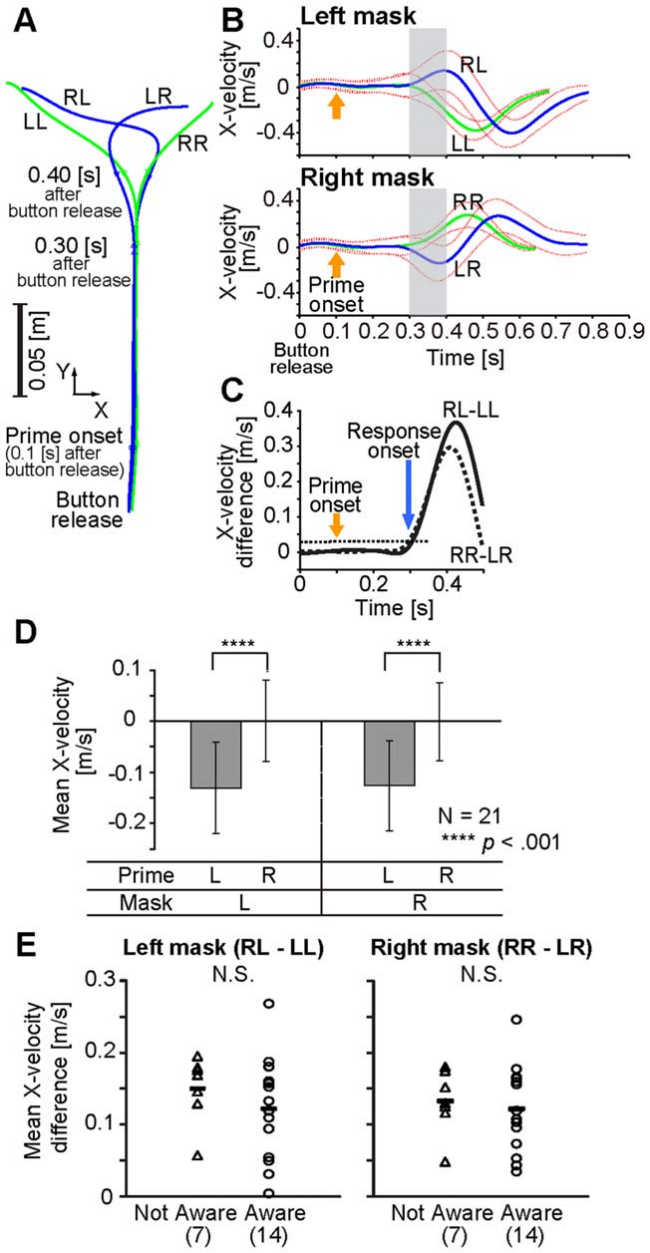
Kinematic properties of reaching movement. (A) Average reach trajectory to the far target of each side in each condition for a single participant. Green and blue curves show trajectories when prime-mask combinations were congruent (RR and LL conditions) and incongruent (LR and RL conditions), respectively. (B) Average X velocity profile in each far-target condition for a single participant. Green and blue curves show the velocities when the prime-mask combination was congruent and incongruent, respectively. Red dashed curves denote ±1 SD of velocities between trials. The data were aligned at the button release. (C) Mean X velocity difference profiles (Solid: RL – LL. Dashed: RR – LR.) calculated from the pooled data to far and near targets. The velocity difference was calculated as the index of the response onset to the prime stimulus. The threshold value (dotted line) was set at 0.03 m/s. (D) Mean X velocity of the 100-ms-time window (calculated from the pooled data to far and near targets) corresponding to the gray area in Fig. 3B (i.e., 200∼300 ms after the prime onset) in each condition. (E) Relationship between the awareness of the association (between the participant’s own performance and the color cue) and the strength of the implicit motor control induced by the invisible stimulus. The difference of mean X velocity in the 100-ms-time window (Fig. 3D) between RL (RR) and LL (LR) conditions for each participant was calculated as the individual strength of the implicit motor control.

Figures 3A and 3B respectively show the trajectories on the horizontal plane and x-directional velocity profiles. When the prime stimulus was incongruent with the mask one (i.e., RL or LR conditions), trajectories shown by blue curves were directed to the opposite direction of the mask stimulus (i.e., the direction of the prime) and then modified to reach for the appropriate direction of the mask triangle. As shown in Fig. 3B, the velocity patterns started to deviate approximately 200 ms after prime onset. Mean response onset to the prime stimulus in reaching for the left target was 209.1 ms (SD = 26.2) and that in reaching for the right target was 204.2 ms (SD = 20.5) (see Fig. 3C). Mean velocity of the 100-ms-time window (from 200 to 300 ms after the prime onset indicated by the gray area in Fig. 3B) in each condition was calculated and an ANOVA with prime (right and left) and mask (right and left) as within-participant factors revealed the main effect of prime (*F*(1, 20) =  99.982, *p*<.001, *partial η^2^* = .833, see Fig. 3D), suggesting the invisible prime affects online motor control. We also confirmed the same significant effects on the X-position at 300 ms after the prime onset. As the strength of the implicit motor control, we calculated each participant’s difference of mean velocities of the 100-ms-time window indicated by the gray area in Fig 3B in both mask conditions (see also Fig. 3D). As shown in Fig. 3E, we found the awareness of the association between their own performance (induced by prime-mask combination) and the color cue did not have any relationship with the strength of the implicit motor control induced by the invisible stimulus (*t*(19) = −.970, *p* = .344 for the left mask, *t*(19) = −.422, *p* = .678 for the right mask).

#### Motor behavior induced by implicit perception correlates with action evaluation

**Figure 4:**
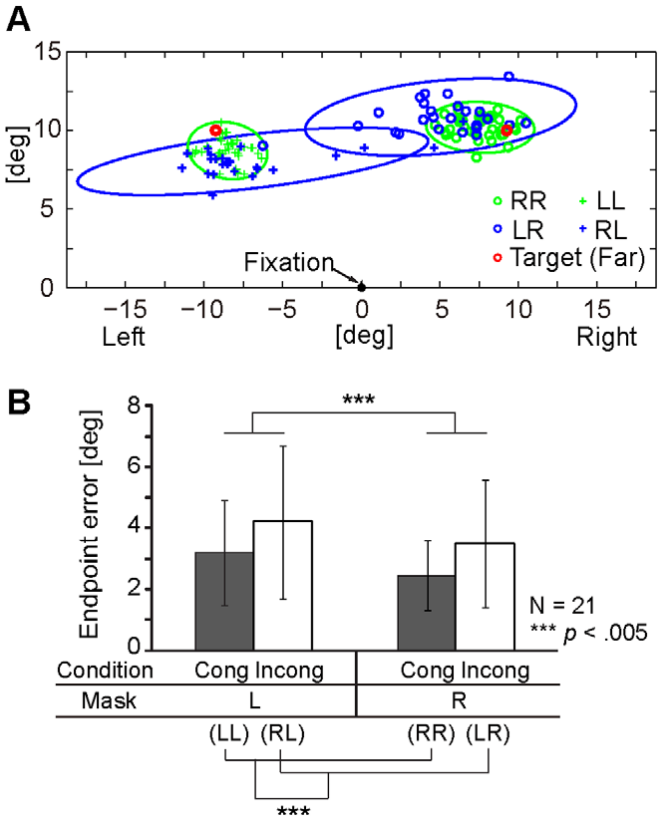
Endpoint error induced by implicit motor control. (A) Endpoint distribution of a single participant and its 95% confidence ellipse in each condition of the far target. Green open circles and crosses respectively denote RR and LL (prime-mask congruent) conditions; blue open circles and crosses respectively denote LR and RL (prime-mask incongruent) conditions. Red open circles indicate the locations of target points. (B) Mean endpoint error in each condition. Data from both target positions (i.e., near and far conditions) were pooled. Error bars indicate the SDs of the endpoint errors across participants. Cong and Incong denote congruent-green and incongruent-blue conditions, respectively.

Fig. 4A shows endpoint distributions for a single participant who reached to the far targets. In the incongruent-blue condition, participants sometimes failed to point to the appropriate target and mistakenly reached to the opposite side. Such cases indicate the effect of the invisible prime triangle was so influential on motor behavior that the participants could not sufficiently modify the trajectory during the reaching movement. In these cases, participants reported they did not know why they performed in such a way, despite their having full awareness of the mask (cue) stimulus direction. Furthermore, some of them reported that such inappropriate performance was due to a loss of concentration during the task.

We evaluated the endpoint error (Fig. 4B), and an ANOVA with mask triangle (right, left) and condition (congruent-green, incongruent-blue) as within-participant factors revealed that the main effect of mask (*F*(1, 20) = 14.717, *p* = .001, *partial η^2^* = .595) and condition (*F*(1, 20) = 13.118, *p* = .002, *partial η^2^* =  567) but no mask×condition interaction (*F*(1, 20) = .058, *p* = .813). There were significant differences between congruent-green and incongruent-blue conditions in both mask triangle stimuli (i.e., right and left).

**Figure 5:**
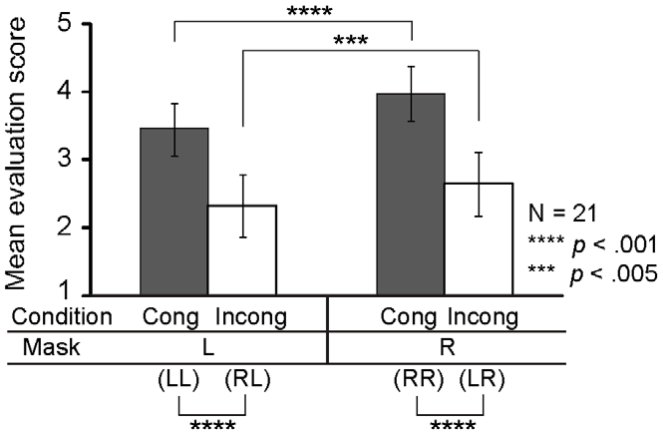
Mean evaluation scores in the practice phase. RR and LL conditions are congruent-green conditions and LR and RL conditions are incongruent-blue conditions. Error bars indicate the SDs of the evaluation scores across participants. Cong and Incong denote congruent-green and incongruent-blue conditions, respectively.

We next calculated the mean evaluation scores (Fig. 5) and found the main effect of mask (*F*(1, 20) =  25.047, *p*<.001, *partial η^2^* = .556), condition (*F*(1, 20) = 122.733, *p*<.001, *partial η^2^* = .860) and the mask×condition interaction (*F*(1, 20) =  5.061, *p* = .036, *partial η^2^* = .202). There were significant differences between congruent-green and incongruent-blue conditions for both mask stimuli and there were also significant differences between mask triangle directions in both conditions (congruent-green, incongruent-blue). These results indicated that participants felt their performance was poorer against their intention in the incongruent-blue condition.

**Figure 6:**
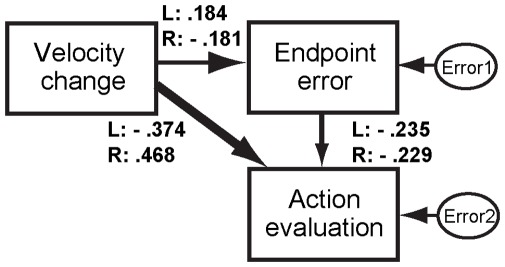
Path diagram in the practice phase. Path analysis was applied to the data of each participant. Velocity change denotes the data of the X velocity of the 100-ms-time window corresponding to the gray area in Fig. 3B (i.e., 200∼300 ms after the prime onset), Endpoint error denotes the endpoint errors, and action evaluation denotes the action evaluation scores reported after each trial. The line width between the variables schematically indicates the strength of the relationship, and each number near the path denotes the mean standardized path coefficient of Mask L and R conditions respectively (see also Table 1).

The results in Fig. 5 would suggest the motor behavior induced by the invisible prime affected the action evaluation. To examine what information (the velocity modification during movement or the endpoint error after movement) is critical for the action evaluation, we applied a path analysis to the hypothesized model in Fig. 6, which shows the mean path coefficients of mask L and R conditions. We found no VIFs over 10 in the analysis of each participant. Table 1 summarizes the standardized path coefficients in the model for each participant. Path coefficients from velocity change to action evaluation showed higher values than those from endpoint error to action evaluation except in the following cases: Participants 7, 17, and 21 in both mask stimuli conditions and participants 11 and 14 in the left-pointing mask stimulus (mask L) condition showed higher path coefficients from endpoint error to action evaluation (marked in superscript a in Table 1). Participant 10 in the mask L condition showed no significant path coefficients (marked in superscript b in Table 1). Participant 11 in the mask R condition showed a value from velocity change to action evaluation comparable to that from endpoint error to action evaluation (marked in superscript c in Table 1).

**Table 1:** Standardized path coefficients of each participant in the practice phase in the model in Fig. 6.

		**Mask L**			**Mask R**		
**Participant**	**Aware**	**Vel→EndPtErr**	**EndPtErr→ActEv**	**Vel→ActEv**	**Vel→EndPtErr**	**EndPtErr→ActEv**	**Vel→ActEv**
1	○	−0.061	−0.138*	−0.675****	−0.314****	−0.075	0.755****
2	○	0.191*	−0.124	−0.676****	0.198*	0.101	0.565****
3	○	−0.020	−0.230***	−0.406****	−0.069	−0.344****	0.553****
4	○	0.026	−0.105	−0.520****	−0.118	−0.258***	0.418****
5	○	0.311****	−0.258****	−0.479****	−0.560****	−0.261****	0.663****
6	○	0.400****	−0.206*	−0.440****	−0.455****	−0.252**	0.428****
7	×	0.430****	−0.548**** a	−0.078	−0.088	−0.650**** a	0.136
8	×	−0.128	−0.254***	−0.504****	−0.118	−0.263***	0.350****
9	○	0.123	−0.043	−0.419****	0.166	−0.127	0.529****
10	○	−0.060	0.077^b^	0.038^b^	0.351****	0.096	0.393****
11	○	0.112	−0.471**** a	−0.145	−0.503****	−0.322*** c	0.320*** c
12	○	−0.113	−0.115	−0.559****	−0.127	0.037	0.692****
13	○	0.453****	−0.288****	−0.584****	−0.641****	−0.309****	0.451****
14	×	0.119	−0.260*** a	−0.138	−0.234***	−0.321****	0.393****
15	×	0.224**	−0.208**	−0.373****	−0.383****	−0.285****	0.557****
16	×	0.437****	−0.204*	−0.339****	0.178*	0.016	0.669****
17	○	0.295****	−0.529**** a	−0.326****	−0.247***	−0.555**** a	0.296****
18	×	0.345****	−0.227***	−0.404****	−0.347****	−0.138	0.443****
19	×	0.323****	−0.068	−0.253***	−0.087	−0.153*	0.348****
20	○	−0.108	−0.184*	−0.417****	0.046	−0.216***	0.538****
21	○	0.567****	−0.550**** a	−0.166*	−0.451****	−0.520**** a	0.337****

Aware: ○ indicates the participants who reported an association between their own performance and the color cue;×indicates those who did not notice such an association. It is noteworthy that all participants showed a higher significant action evaluation score in the congruent-green condition than in the incongruent-blue condition.

Vel: Velocity change; EPtErr: Endpoint error; ActEv: Action evaluation.

a The path coefficients from endpoint error to action evaluation higher than those from velocity change to action evaluation.

b No significant path coefficients in those from velocity change to action evaluation and from endpoint error to action evaluation.

c The comparable path coefficients in those from velocity change to action evaluation and from endpoint error to action evaluation.

**** *p*<.001,

*** *p*<.005,

** *p*<.01,

* *p*<.05.

In summary, 15 participants in the mask L condition and 17 participants in the mask R condition showed a predominant role of velocity change caused by the prime stimulus during movement in the action evaluation, while five participants in the mask L condition and three in the mask R condition showed a predominant role of endpoint error after movement. The results suggest that online motor correction induced by the invisible prime stimulus is more crucial for evaluating motor behavior and that endpoint error is somewhat of a clue for action evaluation but its effect is much weaker than that of motor correction.

### Test Phase

As in the practice phase, only correct trials in the color identification task were analyzed. The mean correct rate of the color identification task in the test phase was 97.5% (SD = 2.2%), indicating participants discriminated the color nearly perfectly as they did in the practice phase.

In this phase, to investigate what information (i.e., online sensorimotor information, endpoint error, or external color cues) predominantly contributes to the evaluation of one’s own motor behavior, we introduced another condition (i.e., the either congruent-blue or incongruent-green condition), in addition to the congruent-green, incongruent-blue, and neutral conditions. Specifically, we wanted to examine whether a color cue irrelevant to one’s own motor behavior would affect the action evaluation or not.

The 21 participants were divided into two groups. For one group, the incongruent-green condition was inserted (incongruent-green group, 11 participants); for the other, the congruent-blue condition was inserted (congruent-blue group, 10 participants).

#### Motor behaviors themselves are not modulated by the external color cue

Table 2 shows mean velocity of the 100-ms-time window in each condition for the incongruent-green and congruent-blue groups. The prime affected online motor behavior in both groups (incongruent-green group: *F*(1, 10) = 24.587, *p*<.001, *partial η^2^* = .711; congruent-blue group: *F*(1, 9) = 49.556, *p*<.001. *partial η^2^* = .846). There were no differences among conditions (incongruent-green group: *F*(2, 20) = 1.860, *p* = .182; congruent-blue group: *F*(2, 18) = 1.497, *p* = .250). We found no prime stimulus×condition interaction (*F*(2, 20) = .495, *p* = .617 for the incongruent-green group; *F*(2, 18) = 1.981, *p* = .167 for the congruent-blue group).

**Table 2:** Mean velocity of the 100-ms-time window in each condition of incongruent-green and congruent-blue groups in the test phase.

	Prime	L			R			Prime
*Incongruent-green group*	Condition	Cong-gr	Incong-bl	Incong-gr	Cong-gr	Incong-bl	Incong-gr	
	Mean	−74.5	−66.7	−61.3	44.7	44.3	46.9	*F*(1, 10) = 24.6
	SD	(102.5)	(101.3)	(97.8)	(89.7)	(79.5)	(90.2)	*p*<.001
*Congruent-blue group*	Condition	Cong-gr	Incong-bl	Cong-bl	Cong-gr	Incong-bl	Cong-bl	
	Mean	−183.0	−178.0	−186.2	−47.6	−36.5	−22.9	*F*(1, 9) = 49.6
	SD	(80.2)	(77.0)	(94.2)	(93.8)	(93.8)	(97.7)	*p*<.001

Cong-gr, Incong-bl, Incong-gr, and Cong-bl denote congruent-green, incongruent-blue, incongruent-green, and congruent-blue conditions, respectively.

Although the values themselves between groups seemed to be different, these differences were due to lower mean values for a few participants in the congruent-green group, not to the difference in the inserted conditions. As shown in Table 3, the mean values in the practice phase with the participants divided into the same two groups as in the test phase were comparable to those in the test phase. The results indicate that motor behaviors induced by the invisible prime stimulus were not influenced by the external color cue.

**Table 3:** Mean velocity of the 100-ms window in each condition of incongruent-green and congruent-blue groups in the practice and test phases.

		*Incongruent-green group*				*Congruent-blue group*			
	Mask	L		R		L		R	
	Prime	L	R	L	R	L	R	L	R
*Practice phase*	Mean	−88.1	33.8	−82.8	30.7	−177.2	−36.0	−174.3	−36.3
	SD	(62.9)	(60.5)	(61.9)	(60.9)	(95.1)	(83.6)	(90.6)	(79.2)
*Test phase*	Mean	−74.5	44.3	−66.7	44.7	−183	−36.5	−178	−47.6
	SD	(102.5)	(79.5)	(101.3)	(89.7)	(80.2)	(93.8)	(77.0)	(93.8)

#### External color cue irrelevant to action itself could be a clue for action evaluation

Table 4 shows mean endpoint errors in each condition for the incongruent-green and congruent-blue groups. In the incongruent-green group, an ANOVA with mask stimulus (right, left) and condition (congruent-green, incongruent-blue, incongruent-green) as within-participant factors revealed the main effect of condition (*F*(2, 20) = 4.532, *p* = .0238, *partial η^2^* = .312) but no main effect of mask stimulus (*F*(1, 10) = .565, *p* = .469) and no condition×mask stimulus interaction (*F*(2, 20) = .741, *p* = .490). There was a significant difference between the congruent-green and incongruent-blue conditions, although the value in the incongruent-green condition was not significantly different from those in the congruent-green and incongruent-blue conditions. In the congruent-blue group, an ANOVA with mask stimulus (right, left) and condition (congruent-green, incongruent-blue, congruent-blue) as within-participant factors found the main effect of condition (*F*(2, 18) = 6.772, *p* = .006, *partial η^2^* = .429), mask stimulus (*F*(1, 9) = 9.236, *p* = .0140, *partial η^2^* = .506) but no condition×mask stimulus interaction (*F*(2, 18) = .636, *p* = .541). Endpoint error in the incongruent-blue condition was significantly larger than in the congruent-green and congruent-blue conditions and there was no difference between the congruent-green and congruent-blue conditions.

**Table 4:** Mean endpoint errors in each condition of incongruent-green and congruent-blue groups in the test phase.

	Mask	L			R			Mask	Condition
*Incongruent-green group*	Condition	Cong-gr	Incong-bl	Incong-gr	Cong-gr	Incong-bl	Incong-gr		
	Mean	2.17	2.75	2.5	1.87	2.43	2.48	*F* (1, 10) = 0.57	*F* (2, 20) = 4.53
	SD	(0.67)	(0.69)	(0.72)	(0.55)	(1.27)	(1.14)	n.s.	*p* = .0238
*Congruent-blue group*	Condition	Cong-gr	Incong-bl	Cong-bl	Cong-gr	Incong-bl	Cong-bl		
	Mean	3.50	4.28	3.48	2.70	3.16	2.49	*F* (1, 9) = 9.24	*F* (2, 18) = 6.77
	SD	(1.95)	(2.99)	(1.92)	(1.23)	(1.75)	(1.29)	*p* = .0140	*p* = .006

Abbreviations are the same as in Table 2.

**Figure 7:**
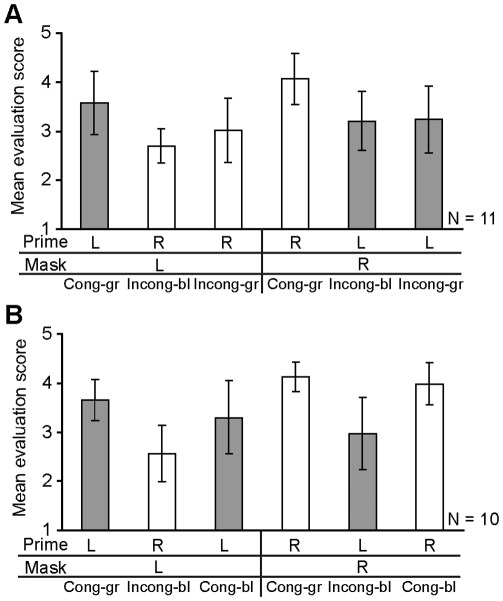
Mean evaluation scores in the test phase. (A) Incongruent-green group and (B) congruent-blue group. Cong-gr, Incong-bl, Cong-bl, and Incong-gr denote congruent-green, incongruent-blue, congruent-blue, and incongruent-green conditions, respectively.

Figs. 7A and 7B show the mean evaluation scores of the incongruent-green and congruent-blue groups, respectively. In the incongruent-green group (Fig. 7A), we conducted a planned *t* test between incongruent-blue and incongruent-green conditions, but we did not find any significant differences in either of the mask conditions (*t*(10) = 2.026, *p* = .070 for the left mask; *t*(10) = .745, *p* = .474 for the right mask). In the congruent-blue group (Fig. 7B), we conducted a planned *t* test between congruent-green and congruent-blue conditions, but we did not find any significant differences in either of the mask conditions (*t*(9) = 1.682, *p* = .127 and *t*(9) = 1.488, *p* = .171 for the left and right masks). These results suggest that the action evaluation seemed to be less commonly influenced by the color external cue across all participants.

**Figure 8:**
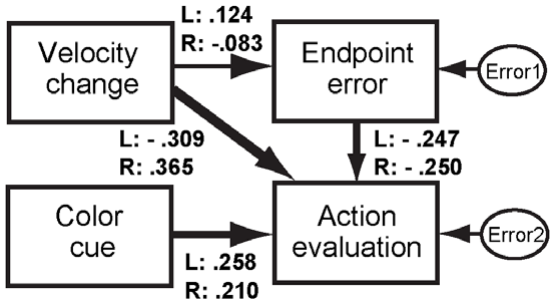
Path diagram in the test phase. In addition to the variables in the practice phase (i.e., velocity change, endpoint error, and action evaluation), a color cue, the color (green or blue) of the fixation point during each trial, was added. The color cue was nominal scale, so we used a dummy variable for the analysis; that is, blue was transformed to 0 and green was transformed to 1. The line width between the variables schematically indicates the strength of the relationship, and each number near the path denotes the mean standardized path coefficient of Mask L and R conditions respectively (see also Table 5).

To examine the details of individual performance, we applied the path analysis to a hypothesized model that added the path from the color cue (green cue = 1, blue cue = 0 as dummy variables) in the action evaluation (Fig. 8) to the model in the practice phase shown in Fig. 6. We found no VIFs over 10 in the analysis of each participant. Table 5 summarizes the standardized path coefficients for each participant and Fig. 8 shows the mean path coefficients of mask L and R conditions. By adding the path from the color cue to the action evaluation, the effect of velocity change was decreased, although it still played a substantial role, and the color cue modulated the action evaluation in some participants. Specifically, the number of the participants who showed the predominant contribution of online motor correction (i.e., velocity change) in the action evaluation decreased (15 -> 6 for the mask L condition; 17 -> 11 for the mask R condition, marked in superscript b in Table 5), while participants who showed the predominant contribution of the external associated color cue in the action evaluation emerged (six participants in the mask L condition; three in the mask R condition, marked superscript c in Table 5).

**Table 5:** Standardized path coefficients of each participant in the test phase in the model in Fig. 8.

			Mask L				Mask R			
	Participant	Aware	Vel → EndPtErr	EndPtErr → ActEv	Vel → ActEv	ColCue → ActEv	Vel → EndPtErr	EndPtErr → ActEv	Vel → ActEv	ColCue → ActEv
*Incongruent-green group*	1	○	−0.020	−0.268***	−0.353****	0.404**** ^c^	−0.135	−0.267***	0.334****	0.466**** ^c^
	2	○	0.339****	−0.162	−0.169	0.707**** ^c^	0.040	−0.068	0.465**** ^b^	0.301****
	3	○	−0.003	−0.340**** ^a^	−0.258*	0.041	−0.315***	−0.417**** ^a^	0.224*	0.056
	4	○	−0.027	−0.023	−0.343*** ^b^	−0.017	0.035	−0.172	0.435**** ^b^	0.050
	5	○	0.362****	−0.404****	−0.501**** ^b^	0.120	−0.377****	−0.397****	0.376****	0.260**
	6	○	0.205*	−0.298***	−0.477**** ^b^	0.080	−0.218*	−0.229*	0.210*	0.244*
	7	×	0.155	−0.466**** ^a^	−0.335****	0.178	−0.155	−0.788**** ^a^	0.031	0.014
	8	×	0.109	0.097	−0.197	0.165	0.153	0.130	0.245* ^b^	0.101
	9	○	0.176	−0.250*	−0.505**** ^b^	0.120	0.056	−0.340****	0.439**** ^b^	0.242*
	10	○	0.061	−0.081	−0.063	0.153	0.321***	−0.002	0.303*** ^b^	0.095
	11	○	0.196	−0.289***	−0.284***	0.320****	−0.047	−0.581*** ^a^	0.418****	0.129
*Congruent−blue group*	12	○	−0.027	−0.264***	−0.406****	0.480**** ^c^	0.000	−0.147	0.351****	0.487**** ^c^
	13	○	0.132	−0.126	−0.510****	0.503****	−0.327***	−0.102	0.646**** ^b^	0.302****
	14	×	0.094	−0.191	−0.289** ^b^	−0.064	−0.235*	−0.076	0.441**** ^b^	0.004
	15	×	−0.053	−0.615**** ^a^	−0.261***	0.264***	−0.091	−0.621**** ^a^	0.353****	0.214***
	16	×	0.431****	−0.432**** ^a^	−0.346****	0.140	−0.039	−0.018	0.430**** ^b^	0.168
	17	○	0.294**	−0.352****	−0.127	0.507**** ^c^	−0.166	−0.560**** ^a^	−0.013	0.333***
	18	×	0.156	0.079	−0.158	0.452**** ^c^	−0.176	0.011	0.297***	0.424**** ^c^
	19	×	−0.074	−0.213	−0.231* ^b^	0.031	0.253*	−0.214*	0.672**** ^b^	0.110
	20	○	−0.286**	−0.165	−0.404****	0.557**** ^c^	0.032	−0.110	0.621**** ^b^	0.315****
	21	○	0.389****	−0.418**** ^a^	−0.269**	0.272***	−0.359***	−0.281**	0.383**** ^b^	0.088

ColCue denotes Color Cue; other abbreviations (Aware, Vel, EPtErr, ActEv) are the same as in Table 1.

a,b,c Largest values among the three path coefficients (i.e., endpoint error → action evaluation, velocity change → action evaluation, color cue → action evaluation) are marked respectively.

**** *p*<.001,

*** *p*<.005,

** *p*<.01,

* *p*<.05.

The results suggest that online sensorimotor information induced by implicit motor control is the main source for the action evaluation and that the endpoint error is a moderate clue in the test phase as well as in the practice one. Furthermore, the path analysis applied for each participant revealed that some participants used the external color cue irrelevant to their own motor behavior as a clue for the action evaluation and that doing so resulted in a biased action evaluation (i.e., misattribution).

## Discussion

The goals of this experiment were 1) to examine the effect of *implicit* perception on online motor control under a condition in which invisibility of the prime stimulus was confirmed by obtaining the perceptual threshold of the prime stimulus intensity for each participant, 2) to investigate how we evaluate our own implicitly emerging motor behaviors induced by the invisible prime, and 3) to identify what information (i.e., internal sensorimotor information, consequent endpoint error, or an external associative cue) is crucial for such action evaluation, or specifically, to verify whether the action evaluation is modulated by an external cue irrelevant to our own motor behavior. We found that the invisible prime affects online control of reaching movement, as shown by previous research (e.g., [30], [31]), and we found a correlation between the action evaluation score and movement induced by the invisible prime, suggesting that monitoring online sensorimotor information is crucial for evaluating our own motor behavior. Furthermore, the results in the test phase suggested the effect of external color cues on action evaluation would emerge in some cases.

### Implicit Online Motor Control Induced by Invisible Visual Stimulus

The visual backward masking paradigm in goal-directed reaching tasks (e.g., [30], [31]) is a good probe for inducing implicit motor control, as Song and Nakayama [29] pointed out. Under such conditions, when the direction of the invisible prime triangle was incongruent with that of the mask triangle, the trajectory initially followed the direction of the prime stimulus (i.e., the direction opposite to the goal) and was then modified to the perceptually instructed (mask) direction (see Fig. 1C; note that the mask stimulus was also a cue stimulus for the reaching direction). Schmidt and his colleagues presented the rapid chase theory [32], [40], [41], where “primes and targets elicit feedforward sweeps that traverse the visuomotor system in strict sequence, without any temporal overlap” [42]. According to this theory, each sweep of the prime and target is able to directly start the independent motor responses in compliance with each stimulus and there is no need for conscious control. This theory could explain such early trajectory deviation induced by the invisible prime stimulus. The present results are in line with the rapid chase theory. The analysis of velocities showed that the effect of the prime began after approximately 200 ms (Fig. 3C). This latency was a little longer than in previous studies that applied the target location change paradigm (e.g., [10], [43]–[45]), in which the response latency was ∼150 ms. This greater latency may reflect the fact that the reaching movement in the current study did not involve a target location shift, but a change in a central cue. It could also be due to slower sensorimotor processing for shape information [46]. Further investigation of subliminal shape information processing in the online control of reaching (cf. [40]) will contribute to our understanding of the interaction mechanism between the dorsal stream and the ventral system (e.g., [29], [47]). In summary, the present study showed that a visual stimulus without perceptual awareness indeed influences online motor control of reaching movement.

### Conscious Monitoring of Implicitly Driven Motor Behavior

We found that the action evaluation was correlated with the motor behavior induced by the invisible prime stimulus (i.e., velocity change induced approximately 200 ms after the prime onset). In the practice phase, participants reported a greater feeling of action smoothness when the prime direction was congruent with the mask one (congruent-green condition) and a lower score when the prime-mask stimulus combination was incongruent (incongruent-blue condition), as shown in Fig. 2A. There are two possible motor clues for action evaluation: (i) afferent information from the motor behavior modulated by the invisible prime and (ii) endpoint error. Path analyses (Fig. 6) revealed that a majority of the participants used appropriately afferent information from their own motor behavior for action evaluation, while the endpoint error also provided a moderate clue for such evaluation. An interesting result of the present study is that, although the endpoint error is a kind of apparent cue, online kinematics change induced by the invisible prime was a more predominant cue for action evaluation than the endpoint error. The appropriate monitoring of online sensorimotor information induced by a non-perceptual stimulus in the present study indicates the dissociation between motor awareness and perceptual awareness as Johnson and Haggard [12] argued, while several studies have suggested that normal individuals are poorly aware of many aspects of their intentional motor acts [27], [48], [49].

A recent patients’ study of anosognosia for hemiplegia demonstrated that motor and premotor areas (particularly area 6) are mainly involved in motor awareness [50]. Haggard and Magno [51] also demonstrated that motor awareness arises somewhere between the primary motor and premotor cortex. While neural bases of motor awareness are assumed to lie in the areas mentioned above, the involvement of the anterior cingulate cortex and the lateral prefrontal cortex in action monitoring has been demonstrated (e.g., [52]). How motor awareness is involved in action evaluation and what neural mechanism underlies such processing are open questions for further investigation.

The number of participants who showed a significant standard path coefficient in the mask L condition was smaller than in the mask R condition. This tendency may reflect the task difficulty due to biomechanical constraints [53], [54]. Participants reported that reaching to the left target was more difficult than reaching to the right one, and the reaching endpoint error in the mask L condition was indeed larger than in the mask R condition (Fig. 4B), so such reports are consistent with the present results. Specifically, the task itself (i.e., reaching to the left target) was difficult, so the online sensorimotor information of the modulated motor behavior did not function well for some participants in evaluating their own action.

In summary, the results demonstrate that we can monitor motor behavior modulated by implicit perception even when such perception makes it impossible for us to detect the source of disrupted motor behavior and that the online sensorimotor information from such modulated motor behavior is fundamental for the action evaluation.

### Biased Action Evaluation Deluded by External Color Cue Irrelevant to Own Motor Behavior

We found that the color cue did not influence the prime effect on the motor behavior itself. As for the action evaluation, the associative color cue did not seem to significantly contribute to the action evaluation (Fig. 7). However, path analyses revealed that some participants used the associative color cue for the action evaluation. In the present study, color cues were associated with the prime-mask congruency in the practice phase, and color cues that were not correlated were inserted in the test phase, so we could test whether the action evaluation depends more strongly on the actual sensorimotor signal or on a visual proxy for the sensorimotor signal. The color cue had high reliability even in the test phase, so participants could have used this color information inferentially for the action evaluation process. The results suggest that internal sensorimotor information makes a large contribution, while proxy color cues make some contribution. Cognitive psychology studies have demonstrated how people attribute and evaluate actions in various situations (e.g., [55]). A recent study demonstrated, in a simple decision task, that participants fail to notice conspicuous mismatches between their intended choice and the outcome they are presented with and that in even such a situation they offer introspectively derived reasons for why they choose the way they do [56].

The task of the present experiment was a motor task rather than a simple decision task, so participants mainly used their online sensorimotor information. But even in such a motor task, the inferential process might be driven by modulating task difficulty. As mentioned above, participants reported that reaching to the left target was more difficult than reaching to the right one. The larger number of participants who used the color cue for action evaluation in the left mask stimulus condition (i.e., six participants) than in the right mask one (i.e., three participants) reflects a decreasing contribution of the online sensorimotor information from the modulated behavior with increasing task difficulty. Specifically, when the task is more difficult (i.e., the target point is left), confidence in the online action monitoring system decreases and the action evaluation is inferred retrospectively from the external color cue and/or the endpoint error. In such a situation, in particular, misattribution to the external color cue irrelevant to one’s own motor behavior occurs. Each contribution of the internal sensorimotor information, external color cue, and endpoint error for the action evaluation is in line with recent studies that examined simple manual action, which suggested that predictive and inferential processes involve the experience of action (e.g., [23], [57], [58]).

In conclusion, the action evaluation is presumably modulated retrospectively by information that is superficially and arbitrarily associated with motor performance, as well as with the fundamental effect of the online sensorimotor information.
